# Re-assessing measurement error in police calls for service: Classifications of events by dispatchers and officers

**DOI:** 10.1371/journal.pone.0260365

**Published:** 2021-12-08

**Authors:** Rylan Simpson, Carlena Orosco

**Affiliations:** 1 School of Criminology, Simon Fraser University, Burnaby, British Columbia, Canada; 2 School of Criminology and Criminal Justice, Arizona State University, Phoenix, Arizona, United States of America; University of California-Irvine, UNITED STATES

## Abstract

Police calls for service are an important conduit by which officers and researchers can obtain insight into public requests for police service. Questions remain, however, about the quality of these data, and, particularly, the prevalence of measurement error in the classifications of events. As part of the present research, we assess the accuracy of call-types used by police dispatchers to describe events that are responded to by police officers. Drawing upon a sample of 515,155 calls for police service, we explore the differences among initial call-types, cleared call-types, and crime-types as documented in crime reports. Our analyses reveal that although the majority of calls for service exhibit overlap in their classifications, many still exhibit evidence of misclassification. Our analyses also reveal that such patterns vary as a function of call- and crime-type categories. We discuss our findings in light of the challenges of the classification process and the associated implications.

## Introduction

In February 2021, police in Morris County, New Jersey received a call about an unattended child at a public park. Upon arrival in the area, police located the deceased bodies of a mother and son in a nearby pond [1]. Just a month later in Surrey, British Columbia, officers were dispatched to a report of a woman in distress inside of a vehicle driven by a male occupant. Upon locating the vehicle’s occupants, officers determined that the distraught woman had attempted to exit the moving vehicle after discovering a spider inside of it [2]. And, again in 2021, police in Milton, Ontario responded to a report of a homicide. Upon arrival at the scene, officers found no evidence of a homicide, but instead indications of a swatting incident: a prank call made to the police with the intent of soliciting an unsubstantiated police response [3].

Events can quickly change and evolve, as can the details surrounding such events, over the course of a call’s lifespan. Events can change in both risk and substance: from low risk to high risk, from high risk to low risk, and from one call-type to another. Although the examples highlighted above represent some egregious cases, more mundane cases may be much more common. Events that are initially reported as robberies may be thefts, events that are initially reported as impaired drivers may actually be drivers in medical distress, and events that are initially reported as traffic collisions may just be abandoned vehicles. In many cases, requests for police service are convoluted and unclear, and caller-provided information is ambiguous and nebulous, making initial classifications of such requests particularly challenging.

The challenge of classifying events into pre-defined call-types is highlighted by the very fact that most police agencies actually include ambiguous call-types, including “Abandoned 911,” “Alarm,” “Suspicious Circumstance” and “Unknown Trouble,” in their list of pre-defined call-types to be used by dispatchers (specifically those working in the capacity of call-takers) when initially classifying events. In many of these instances, these call-types need to be updated as new information is received by police, including via officers’ attendance, given that the initial call-type itself offers little substance about the nature of the event. For example, the “Unknown “Trouble” call-type reflects instances where police are requested, at least implicitly, but for a reason not known at the time of the initial call generation. The “Suspicious Circumstance” call-type reflects instances where someone believes a crime has occurred or may be about to occur, but such crime is not obvious or cannot be confirmed until an officer responds. And a similar logic applies for events classified using the “Alarm” call-type, most of which are false, but on some occasions can alert police to a robbery or burglary in-progress.

The ambiguity inherent in calls for service exhibits important implications for both operations and research. From an operational perspective, ambiguity can mean that events are misclassified by dispatchers, and in turn misinterpreted by responding officers, which could result in problematic decision-making by police (e.g., see [4–6]). For example, if officers believe that they are responding to a call for service involving a weapon based upon its initial call-type, then they may be more likely to respond with force upon arrival. Similarly, if officers think that they are responding to a call for service where a crime has occurred, then they may be more likely to make an initial detention upon arrival.

From a research perspective, ambiguity can mean that analyzing calls for service data may misguide one’s understanding of police work and the demands in which the public places on police (e.g., see [5, 7–12]). Misclassified events could result in everything from the misidentification of hot spots to a misunderstanding of the public’s requests for police and their associated willingness to cooperate with police. Indeed, if calls for service are not accurately classified via their respective call-types, then calls for service data could produce an image of public demand that may not reflect reality. If such misclassifications over- or underrepresent particular categories of events, including specifically non-crime-related requests, then it may hinder scholars’ ability to effectively assess the effects of social events or changes in policing on changes in both public and police activity.

The implications of misclassifications are thus vast. As part of the present research, we contribute to this discussion by exploring classification decisions among a sample of more than half a million calls for police service in Chandler, Arizona. Informed by both existing literature and our professional experience as police dispatchers, we empirically assess the accuracy of call-types used by dispatchers to describe events that are responded to by officers. Our analyses reveal that although the majority of calls for service exhibit overlap in their classifications, many still exhibit evidence of misclassification. Our analyses also reveal that these classification patterns vary as a function of call-type (e.g., “Alarm” versus “Unknown Trouble”; in-progress versus not in-progress) and crime-type categories (e.g., “Theft” versus “Assault”). We discuss our results with respect to measurement error and its relevance for both operational responses by police and the use of calls for service data by researchers.

## Background

In 1968, the criminal justice landscape was transformed by the advent of the 911 system [13]. Allowing residents to contact police via telephone, the 911 system made emergency services more accessible than ever before. Now serving as the emergency response system for most of the modern world, the 911 system (or equivalent thereof) allows people in crisis to quickly request emergency services. The convenience and ease of access of 911 has increased the reliance that citizens and police now exhibit on each other [13, 14]. It has also increased the overall number of contacts between the public and the police, leading to growing numbers of calls being filtered through the 911 system each year. Given that dispatchers are tasked with managing 911 calls and initiating the associated response, their role has become increasingly important over time.

### Dispatchers and the 911 system

Dispatchers tasked with answering 911 calls (i.e., call-takers) receive, interpret, classify, and prioritize calls for service (e.g., [5, 7, 8, 10, 11, 15–18]), a process which they are expected to complete accurately, reliably, and quickly in order to initiate a response and then field the next call. As part of their role, they create formal calls for service in the computer-aided dispatch (CAD) system: an electronic repository that is used primarily for operational purposes, but is also linked to the respective police agency’s records management system. Though highly understudied in the criminological literature [5], a recent surge in studies surrounding the actions and impacts of dispatchers (e.g., see [6, 7, 10, 19, 20]) have illuminated both the interpretive and discretionary nature of dispatch work and the impact that dispatchers’ decisions can have on the criminal justice system. As gatekeepers of their agencies [5, 10, 12, 15, 21], actions taken by dispatchers can impact how operational resources are distributed [22, 23] and influence subsequent responses by other actors in the criminal justice system [4, 6, 24]. Because of such effect, dispatchers exhibit at least some influence on almost all calls for service at some point in time [5, 10].

#### Dispatcher classifications

As the first point of contact between citizens and police officers, dispatchers are responsible for ensuring that help is provided where help is required. In order to achieve this responsibility, dispatchers working in the capacity of call-takers must interpret often ambiguous and erratic information from callers, piece together details about events, assign events to call-types, and prioritize the police response to such events based on perceived urgency and possible risk. This process of receiving, handling, and classifying calls into specific call-types is both complex and challenging for several reasons.

First, the classification process must often be completed under much stress and time pressure [5, 18]. Calls frequently need to be generated and classified quickly so that dispatchers can send officers and then field the next call. Such time pressure can mean that information is lost or sometimes even overlooked during the summarizing process. A caller may provide three to four minutes (or more) worth of description about an event, or a series of events, that a dispatcher must then classify into a one- or two-word pre-defined call-type. This can force a dispatcher to base their initial decisions about an event on very limited details. Even though these limited details may arguably exhibit greater accuracy for some events, like those in-progress (given that such events typically present a more obvious need for attendance), there always exists the possibility of misclassification.

Second, callers may provide their information to dispatchers under less than ideal circumstances [16]. Callers may be in heightened or altered emotional states. Some may not fully cooperate with dispatchers for a number of reasons, most of which do not reflect malintent, including injury, trauma, and/or intoxication. Callers may also not provide their contact information or request no follow-up from police prior to disconnecting with dispatchers, thereby limiting the ability for dispatchers to clarify and/or confirm pertinent event details. This kind of context can make collecting and synthesizing the already fragmented details frequently provided by callers difficult [5, 7, 25, 26].

Third, the information that callers do provide is “often ambiguous, nebulous, unclear, suspicious, and/or chronologically disordered” ([5, p. 4]; also see [16, 27–29]). The reason that police are being requested may not be immediately obvious to the dispatcher. And, even if it may seem obvious, the request may be for multiple different reasons. For example, a caller could make a complaint about disturbing noise originating from a house party that is occurring contrary to local bylaws. A caller could request that police conduct a wellness check on their friend who they claim is mentally ill, the victim of a burglary, and the suspect in a theft. Even with more than 100 different call-types, it is possible that an event may not fit well into any single call-type or fit into too many call-types to pick just one.

Fourth, calls for service can evolve over time as additional details emerge from the primary caller, secondary callers, officers on-scene, and/or other witnesses at the location of the event [7, 28]. Events may not always be as clear-cut as a single, unchanged call classification would allow, particularly in the context of ambiguous calls. For example, an event that may start as a suspicious person loitering outside of a residence could later become a violation of an order of protection which could later become a domestic disturbance. Conversely, an event that may start as a person attempting to burglarize a residence may be cleared as a non-event when officers determine that the alleged suspect is actually the homeowner locked out of their own residence. Call-type classifications can be continuously shaped by both citizens and police.

Finally, there is little consistency with regard to the training of dispatchers and their approach to classifying events [13, 30]. As far as we are aware, there is little to no standardized or systematic method of triaging calls for service, classifying events as particular call-types, or prioritizing calls beyond broad recommendations. Without systematic guidelines, the responsibility of designing appropriate classification systems rests upon the shoulders of individual communications centers’ training staff. As a result, the content of training as well as the depth and quality of such training can vary considerably by agency. For such reason, dispatchers may interpret and process the same events differently depending upon the specific set of training they received from their specific agency/trainer, which can make analyzing classification decisions across staff and agencies challenging.

The ambiguity and complexity of caller-provided information and the associated challenges of making sense of such information thus lends itself naturally to a concern about classification, and, particularly, the potential for misclassification. If events cannot be easily classified into pre-defined call-types, then it is possible that events can be misclassified at their initial point of generation. For example, events that may initially be reported as burglaries might later be defined as trespassing. Events that may initially appear to be criminal in nature may eventually turn out to be civil in nature. And events that involve suspicious persons or vehicles may not actually be suspicious at all upon arrival by officers.

### Measurement error

The shortcomings of administrative data are no stranger to existing literature (e.g., [31–34]), and calls for service records have understandably been among the most contentious data sources for researchers and practitioners alike (e.g., [5, 9, 11, 12]). While the nuanced nature of calls for service data has not been examined extensively in the specific context of dispatchers, existing discussions surrounding their role in call classification highlights the complexity of the dispatching process and its implications for data quality [35]. Many of the criticisms among this domain regard the prevalence and magnitude of measurement error that may exist in calls for service data.

Assessing measurement error in calls for service data has been the subject of some past empirical attention. In their seminal study, Klinger and Bridges [9] assessed misclassification errors, specifically false negatives (i.e., classified as non-criminal but determined to be criminal), false positives (i.e., classified as criminal but determined to be non-criminal) and crime misclassifications (i.e., classified as one crime-type but determined to be another), in neighborhoods across the United States. Their results revealed that vandalism calls had the most accurate call classifications (81%), trespass calls had the least accurate call classifications (55%), and robberies were the most likely to be misclassified (16%).

A related examination of assaults and burglaries in Boston by Nesbary [11] similarly found that much variation exists among call-type classifications and priority levels assigned by dispatchers. Nesbary [11] argued that call-takers’ assessments of in-progress events most significantly impacted classification decisions, and those incidents initially considered more serious were most likely to be downgraded to a lower priority or different classification after dispatch. For example, their analysis of assaults revealed a nearly 80% likelihood that the call would be reclassified as another call-type. A similar set of findings regarding misclassifications were observed in San Antonio by Varano and colleagues [12].

Though these foundational studies are tantamount to our current understanding of call classification, we recognize that much has changed in policing in the last two decades that may have influenced classification accuracy. New social demands have been placed on police, and new call-types have been added to the possible list of call-types to be used by dispatchers. Visiting police stations and flagging down police officers to directly report incidents have also largely been replaced by the convenience of calling the police via telephone. It is therefore possible that the amount of measurement error present in calls for service data may have changed over time. On one hand, the ability of dispatchers to select from a larger list of pre-defined call-types should theoretically allow them to more accurately classify events into more tailored call-types, and hence enhance classification accuracy. However, on the other hand, the opportunity to select from a larger list of call-types could actually introduce even more room for ambiguity in the classification process, which in turn could make the classifications of events more inconsistent and thereby less accurate. With more nuance comes more opportunity for interpretation, and more interpretation could potentially induce greater measurement error.

## Overview of the present research

Rooted in existing work regarding police dispatchers and their implications for operations and research, we assess measurement error in calls for service data using a sample of more than half a million calls for police service in Chandler, Arizona. As part of our analyses, we explore the differences, or lack thereof, among events’ initial call-types, cleared call-types, and crime-types as documented in crime reports. In doing so, we assess the overlap between classifications of events by different policing actors at different parts of the policing process and highlight the implications of such classifications for policing affairs.

## Data and methods

### Setting

The present research examines data for Chandler, Arizona. Chandler is a 64-square mile suburb of Phoenix, located in Maricopa County, with a population of approximately 260,000 people [36]. The city falls below the national poverty rate (7.6% as compared to 10.5%), with a median household income of $82,925. It has a violent crime rate of 228 per 100,000 people and a property crime rate of 2,071 per 100,000 people [37]. These crime rates are both lower than neighboring Phoenix, which also has a lower median income and higher poverty rate than Chandler. Chandler is policed by the Chandler Police Department (CPD), which provides public safety services for the city. The CPD employs approximately 330 sworn officers and 170 civilian employees.

### Data

The present research utilizes police calls for service and crime report data for 2013–2019. All data were publicly downloaded from the CPD’s Open Data Portal and merged via their shared file number (N = 1,058,607 events). Given our focus on the interpretive work of dispatchers working in the capacity of call-takers, we dropped all events that were not believed to be initiated by a telephone call to the police (n = 335,839). The majority of these dropped events were generated as “On View” (n = 285,247) by officers and reflected traffic stops (n = 225,809) or subject stops (n = 16,746). The remaining dropped events of this genre were received via the following methods: “Desk Officer,” “Recurring Call,” “Text to 911,” or “Zip Whip Text Message.” We also dropped all events that were classified as call-types that, again, did not appear to be initiated by a telephone call to the police (n = 38,580 events associated to 23 different call-types). These call-types included “Humane Society,” “ID Tech Call,” “License Plate Reader,” and “Police Info.” We note here that regardless of call-type, we retained events that were initiated by 911 and non-emergency calls given that emergencies can still be reported via the non-emergency line and it is often the same dispatchers who answer both 911 and non-emergency calls. Relatedly, we dropped all events that were received by police but then immediately transferred to the fire department (n = 96,857).

Given our interest in assessing files that had both dispatcher and officer involvement, we also dropped all events that were cleared with the code, “Cancel,” (n = 52,109). Finally, we dropped all events that were missing initial or cleared call-type information (n = 20,067 events) as such information was required in order for us to conduct our analyses. This resulted in a final sample of 515,155 telephone-initiated events across seven years, which we analyzed as part of our work.

#### Call-type categorizations

As part of the present research, we sought to examine the accuracy of call-types used by police dispatchers to describe events that are responded to by police officers. However, without both listening to the initial call with the dispatcher and simultaneously responding to such call with the officer, it can be difficult to assess the accuracy of these initial classification decisions. In lieu of such observation (which exhibits many challenges itself), we thus compare each event’s initial call-type (i.e., generated by dispatchers) with its cleared call-type (i.e., generated by responding officers). By comparing these two call-types (as well as the crime-type as documented in the crime report, if applicable), we are able to assess overlap in classifications of events by the dispatcher handling the call and the officer responding to that call.

In their raw form, our sample of 515,155 telephone-initiated events were associated with 109 different call-types. This volume of call-types was not feasible to analyze from either a practical or theoretical perspective. In order to thus manage such volume, we grouped each call-type into a broader category that effectively captured their shared theme. For example, we grouped all of the different alarm-related call-types into the shared category of “Alarm” and all of the different traffic collision-related call-types into the shared category of “Traffic Collision.” This resulted in a final list of 30 different call-type categories. This list was more manageable from a practical perspective and more intuitive from a theoretical perspective, and, therefore, we retained these categories for our analyses. See Table 1 for a list of all call-type categories and their descriptive statistics by initial and cleared call-type.

**Table 1:** Call-type categories and their descriptive statistics.

		Initial Call-Type		Cleared Call-Type
Event Category	# Call-Types	# Events	% All Events	% All Events
Suspicious Circumstance	4	54,306	11	11
Alarm	6	50,733	10	10
Disturbance	5	44,975	9	10
Traffic Collision	12	36,839	7	7
Theft	6	36,001	7	6
Domestic Disturbance	1	31,473	6	4
Wellness Check	2	26,463	5	5
Traffic Complaint	8	26,107	5	5
Assist General Public	5	25,044	5	10
Assist Other Agency	5	23,354	5	5
Burglary	3	18,786	4	3
Abandoned 911	3	17,992	3	3
Abandoned Vehicle	1	11,434	2	2
Fraud	2	10,887	2	2
Bylaw	4	9,887	2	2
Other	10	9,800	2	2
Mental Health	3	9,310	2	1
Assist Fire Department	2	9,036	2	2
Unknown Trouble	1	8,514	2	0
Missing Person	2	8,262	2	1
Mischief	2	8,080	2	2
Assault	2	6,903	1	1
Warrant	1	6,832	1	2
Property	2	6,478	1	1
Drugs	1	5,373	1	1
Weapon	4	4,120	1	1
Child	3	3,887	1	1
Sex Offense	4	3,156	1	1
Robbery	3	803	<1	<1
Dead Body	2	320	<1	<1
**30 Categories**	**109**	**515,155**	**100%**	**100%**

### Analytic strategy

In our first set of analyses, we examined the overlap between initial and cleared call-types for all call-type categories as described above. A classification was assessed as accurate if the initial and cleared call-type categories matched. For example, a call for service that was both created as a “Traffic Collision” and cleared as a “Traffic Collision” was assessed as an accurately classified event. In contrast, a call for service that was created as a “Traffic Collision” but cleared as an “Abandoned Vehicle” was assessed as a misclassified event. As part of this set of analyses, we also explored patterns in classification accuracy by specific call-type categories, including, for example, if the event was believed to be in-progress or not. Assessing whether an event was in-progress, though, was very difficult using call-type information alone. Although some call-types clearly regard in-progress events (e.g., “Burglary In-Progress”), most do not. We therefore classified events as being in-progress if the event had to be in-progress in order to warrant the use of the specific call-type. For example, an alarm must be ringing to initiate a call for service for an “Alarm” (otherwise it would be classified as a different call-type, like a “Burglary” if one occurred). We did not use the priority variable as an indication of in-progress status given that priority classifications also exhibit many challenges and exploring those challenges was beyond the scope of the present research.

In our second set of analyses, we then included crime report data (if applicable) to further assess the accuracy of the initial classifications made by dispatchers. As part of these analyses, we defined an accurate classification as not just overlap between the initial and cleared call-type categories, but also between such call-type categories and the associated crime-type as documented in the crime report. For example, a call for service that was both created and cleared as a “Burglary” *and* had an associated crime report of a “Burglary” was assessed as an accurate classification. A call for service that was both created and cleared as a “Burglary” but either did not have an associated crime report or had a crime report for a different type of crime was assessed as an inaccurate classification. We treat the absence of a crime report for an event accurately cleared as a crime-type as an inaccurate classification for this set of analyses given that such decision reflects a disconnect in the way in which the initial dispatcher and responding officer interpreted the event.

Before proceeding to a description of our results, we first note three caveats regarding our analytic approach. First, we recognize that our work is inherently exploratory in nature. This was expected given the focus of our research and the limited work that has explored similar research questions. Second, we acknowledge that our definition of accuracy may be contested. As alluded to throughout our introduction, it is possible to classify events as multiple different call-types and to place call-types into multiple different thematic categories. For example, an unknown person acting erratically in the yard of a residence could be classified using the call-types of “Trespass” or “Suspicious Person” or “Wellness Check” or “Peeping Tom” or “Prowler,” which in turn could be broadly categorized as a “Suspicious Circumstance” or “Wellness Check” or “Sex Offense.” With that being said, we drew upon existing literature and our professional experience as police dispatchers to try and categorize each call-type to the best of our ability and suggest that such categorizations be the subject of future research. Finally, our analytic approach is linear, insofar that we started with the initial classification of an event by the dispatcher and then moved forward (i.e., from initial call-type category to cleared call-type category to crime-type as documented in the crime report, if applicable).

## Results

### Aggregate results

Our analyses reveal that approximately 85% of all events were cleared as the same call-type category in which they were generated. This classification accuracy rate was relatively constant across years, although appeared to increase very slightly over time. This rate also increased to 94% when excluding events that were cleared without formal officer-citizen contact.

On that note, one could make the argument that we should have excluded events without formal officer-citizen contact from our analyses. However, we elected to retain them for two reasons. First, officers can still make judgments regarding the information about calls for service that is provided to them by dispatchers even without formal contact with anyone directly involved in the event (e.g., identifying an error in how a crime was defined by a dispatcher). Second, for some events, no contact is a meaningful outcome: for example, if a suspect was not located while making patrols for an event classified as a “Suspicious Person.” Retaining these events thus honored the structure of these data: a call is still generated and cleared with a classification even if the officer is unable to make formal contact with the involved parties.

Accuracy rates for classifications, however, were not consistent across all call-type categories. As shown in Table 2, some call-type categories exhibited much greater classification accuracy than other call-type categories. For example, the call-type category of “Alarm” exhibited the greatest overlap between initial and cleared call-types (99%). This was expected given that decisions to create calls for service related to this category are largely organizational as opposed to discretionary, and therefore there is less room for dispatcher interpretation (for related discussions, see [8, 10]). The call-type categories of “Abandoned Vehicle” (98%), “Assist Other Agency” (98%), and “Warrant” (98%) also exhibited much overlap. On the other hand, the call-type category of “Unknown Trouble” exhibited no overlap (0%), as expected for the reasons discussed earlier in our article. The call-type categories of “Assault” (51%), “Domestic Disturbance” (58%), “Mental Health” (65%), and “Child” (73%) also showed little overlap between initial and cleared call-types.

**Table 2:** Call-type categories and their classification accuracy rates.

Event Category	% Accuracy Between Initial and Cleared Call-Type Categories
Alarm	99
Abandoned Vehicle	98
Assist Other Agency	98
Warrant	98
Dead Body	95
Abandoned 911	93
Traffic Collision	93
Assist General Public	92
Traffic Complaint	92
Bylaw	89
Fraud	89
Property	89
Suspicious Circumstance	87
Theft	87
Disturbance	85
Mischief	85
Assist Fire Department	83
Wellness Check	83
Missing Person	82
Sex Offense	81
Burglary	80
Drugs	80
Robbery	80
Weapon	80
Other	75
Child	73
Mental Health	65
Domestic Disturbance	58
Assault	51
Unknown Trouble	0

Analyses of some of these highly misclassified call-type categories offer insight into the potential explanations for such misclassifications. For example, misclassified events of the “Unknown Trouble” category (n = 8,514) were most often cleared as a “Disturbance” (22%), “Assist General Public” (21%), or “Suspicious Circumstance” (13%). Misclassified events of the “Mental Health” category (n = 3,302) were most often cleared as a “Wellness Check” (31%), “Assist General Public” (25%), or “Assist Fire Department” (24%). These findings help to explain some of the discrepancies in the numbers of events by initial/cleared call-type categories presented in Table 1. These findings also suggest that ambiguous calls at the outset are often cleared as arguably ambiguous and non-crime-related call-types at their closure. Indeed, of such misclassified events of these categories, most did not result in a criminal offense as assessed by the crime report data. These findings reaffirm the diversity of calls in which contemporary patrol officers respond to as part of their work and the difficulty in categorizing such calls into pre-defined call-types (e.g., even with so many different call-types, vague call-types still exist to capture events that otherwise do not fit into any other call-type). It is possible that these kinds of events do not present any “real” perceived need for police (at least in a traditional sense), but officers still respond nonetheless, which makes categorizing them difficult: if there is no obvious reason for police to respond, then there may be no obvious call-type to use to describe the event. These findings are consistent with related literature which has found that many calls to the police do not regard crime (e.g., [8, 10, 15]).

When accounting for the emergent nature of call-types, we find that events initially classified as being in-progress exhibited greater classification accuracy (93%) than those not likely to be in-progress (83%). These findings speak back to the potential relevance of ambiguity for classification. If events are in-progress, then they may present a more obvious need for police attendance, and such obvious need may in turn make the events easier to classify (although this may not always be the case, see [11]). In order to further explore this effect, we next turn to our analyses of call-type categories that specifically regard crime.

### Results by crime-type

When analyzing events of our crime-related call-type categories (n = 79,102), we find reasonable overlap in classifications between initial and cleared call-types (mean = 81%). For example, the accuracy rate for “Theft” was 87%, “Mischief” was 85%, “Sex Offense” was 81%, and “Burglary,” “Drugs,” and “Robbery” were 80%. “Assault” exhibited the lowest accuracy rate of 51%.

When we include crime-type information obtained via the crime report data as part of our assessments, however, we observe more complex results. As shown in Table 3, the accuracy rates for initial and cleared call-type categories are higher than the accuracy rates for accurately classified call-type and crime-type categories, with the exception of “Assault” (which may be in part due to the complicated nature of assault classifications, specifically with respect to domestic violence). The highest accuracy rates across all three classifications were observed for “Burglary” and “Robbery” and the lowest rate was observed for “Drugs.”

**Table 3:** Crime-related call-type categories and their classification accuracy rates.

Event Category	% Accuracy Between Initial and Cleared Call-Type Categories	% Accuracy Between Accurately Classified Call-Type and Crime-Type Categories
Assault	51%	72%
Burglary	80%	73%
Drugs	80%	47%
Mischief	85%	63%
Robbery	80%	73%
Sex Offense	81%	68%
Theft	87%	67%

For example, 80% of events initially classified by dispatchers as a “Burglary” were cleared by the responding officer as a “Burglary.” 73% of such events cleared by the responding officer as a “Burglary” resulted in a crime report that classified the offense as a “Burglary” (the remaining events either did not result in a crime report or the crime report documented a different crime-type), suggesting that a “real” burglary likely occurred at that event. In the context of drug offenses, 80% of events initially classified by dispatchers as “Drugs” were cleared by the responding officer as “Drugs.” However, only 47% of such events cleared by the responding officer as “Drugs” resulted in a crime report that classified the offense as “Drugs” (the remaining events either did not result in a crime report or the crime report documented a different crime-type), suggesting that a “real” drug-related offense likely occurred at that event. The same interpretation applies for the remaining crime-related call-type categories of interest.

These findings raise several important points. First, not all events that apparently relate to crime, as initially classified by the dispatcher, are cleared as crime-related call-type categories. Moreover, even if such events are cleared as crime-related call-type categories, not all events are cleared as the same crime-type in which was initially classified by the dispatcher (e.g., initial call-type category of “Burglary,” but cleared call-type category of “Theft”). This suggests that there may be miscommunication from the caller about the event or the dispatcher handling the call may mistake or misunderstand the nature of the event or the associated definition of the criminal offense.

Second, not all events that are cleared by officers as being crime-related result in official crime reports or result in crime reports for different kinds of crime (e.g., cleared call-type category of “Robbery,” but crime-type category of “Burglary”). This suggests that officer discretion, for example, may impact the decision to proceed with formal charges or investigational follow-up, which could lead to crime-related events not resulting in crime reports. Indeed, one would theoretically expect nearly perfect overlap between the cleared call-type category and the crime-type category for these alleged crime-related events: if a crime occurred and therefore the event was cleared as such crime-related call-type category, one would expect the same kind of crime report for that event to follow. However, again, this was not observed in many cases, especially for the crime categories of “Drugs” and “Mischief.” This speaks back to why we included events that were cleared as crime-types without crime reports when calculating these accuracy rates. Although the rates would have improved had we excluded such cases (in part because the differences in numbers being compared across classification points would have been smaller), we believe that the decision to not generate a formal crime report for an event created and cleared as a crime-type indicates that the event was still initially misclassified. In such cases, there exists a potential disconnect between the way in which the dispatcher initially perceived the event (as a legitimate crime worthy of formal documentation) and the way in which the responding officer perceived the event (an event not worthy of further documentation).

These particular findings could also suggest that new information may come to light following clearance of the call for service that could change the officer’s decision about what kind of crime may have occurred at the event (if any). In these kinds of cases, the crime report data would arguably reflect the most accurate description of the event as opposed to the cleared call-type category. For example, the event might appear as a “Burglary” at the time of clearance, but after some further investigation, could be reclassified as a “Theft.” Testing this question, however, would require more qualitative analyses of each event of this nature, which was beyond the scope of the present research.

Finally, and more broadly, only a modest percentage of all events that were initially classified as crime-related call-type categories appear to actually reflect “real” crimes as defined by officers. In this vein, using counts only from calls for service data could inflate the perceived prevalence of some types of crime (e.g., there were more calls for service initially classified using the call-type categories of “Burglary,” “Mischief,” “Sex Offense,” and “Theft” in our sample than there were crime reports for these crime-types). Using these data could also distort the perceived type of crime that may be occurring in a jurisdiction. Although our analyses rest upon several assumptions about the nature of classifications, they still suggest some important discrepancies regarding what kinds and how much crime is occurring in a jurisdiction.

## Discussion

The primary means by which the public can solicit the assistance of the police is via a call for service. All calls to the police must be handled and interpreted by a dispatcher working in the capacity of a call-taker. As part of the call-taking process, dispatchers must decide if a formal call for service is to be generated in the CAD system for response by officers, and if so, how to classify it. As suggested throughout our article, the decision to classify calls for service into pre-defined call-types presents many challenges. Calls are handled under much stress and time pressure. Dispatchers, who receive varying degrees and types of training, must sort through bouts of often ambiguous and nebulous information to make sense of requests that are presented to them by callers under difficult conditions. Calls can change over time, and the reasons for requiring a police response can vary as a call evolves.

Despite these challenges, though, we find that most calls for service still appear to be classified accurately, insofar that they are cleared as the same call-type category in which they were generated. The overall accuracy rate across our 515,155 telephone-initiated events was 85%. The highest accuracy rate was observed for the call-type category of “Alarm.” The lowest accuracy rate was observed for the call-type category of “Unknown Trouble.” These findings are promising, as they indicate that for the majority of events, the initial call-type should at least theoretically reflect the nature of the request. This is important for two reasons. First, researchers who use only initial call-type information may still be able to test their research questions with reasonable confidence about the substance of events included in their analyses. Second, initial call-types are most often what are publicly released by police and available for analyses by researchers (a point which we discuss in greater detail in our implications section below). This accuracy rate is still not perfect though, suggesting that many calls for service are still potentially being misclassified, and hence producing discrepancies in event numbers by initial versus cleared call-type.

Assessing the accuracy of classifications, however, is arguably as challenging as the very process of initially classifying events. Without actually listening to each call as it is received and processed by dispatchers and simultaneously responding to such calls with the associated officers, it is difficult to quantify the overlap in dispatcher and officer classifications, especially at a large scale like done as part of the present research. We thus employed a unique approach to try and post hoc assess the accuracy of initial call-type classifications made by dispatchers working in an urban police agency in Arizona.

Our results revealed several important observations. First, ambiguity appears to exhibit at least some effect on the classification accuracy of events. Consistent with our theorizing, call-type categories that are more obviously ambiguous generally produced less overlap between classifications than call-type categories that are less ambiguous. For example, events that are classified using the ambiguous call-type of “Unknown Trouble” are typically classified that way because of incomplete event details or time constraints that prevent the dispatcher from gathering more concrete details about the event before generating the formal call for service in the CAD system. Once additional information (if any) has been gathered by supplemental conversations with callers and/or information from officers, the call-types for these calls are then typically updated in their cleared form to represent a more accurate classification (and hence one that can be different from the initial classification).

On that note, we recognize that this process of updating call-types from generation to clearance unfortunately does not happen all of the time (although it did for the aforementioned call-type of “Unknown Trouble”). Among our results, we observed that many of the events initially classified using ambiguous call-types were still cleared using similarly ambiguous call-types, indicating that no new information could be obtained, or even with the new information, the request was still too ambiguous to use a more nuanced call-type. For example, 87% of events initially classified as a “Suspicious Circumstance” were cleared as the same call-type category. This observation further underscores that the associated request for police may not have been obvious or explicit in nature at its outset and therefore was still difficult to classify at its closure (for a related discussion, see [38]). It is also possible that officers may not electronically update the cleared call-type as part of the clearance process, which could speak to performance issues and suggestions for better training to ensure compliance in the updating of call-types at the time of clearance.

We also find that the emergent nature of calls can impact classification accuracy: events initially classified using in-progress call-types were less likely to be misclassified than those classified using not in-progress call-types. This was expected given that calls which are initially classified using in-progress call-types are usually categorized in that manner because of specific details provided by a caller that alert the dispatcher to the obvious need for police attendance. Calls of this genre can result in an expedited response by officers, which may sometimes include responding with lights and sirens (i.e., “code 3”), specialty units, and a field supervisor. Given the implications of these additional resources, it is expected that they only be deployed in instances where they can be justified, namely for events that more clearly articulate the need for them.

Finally, we find that events initially classified using call-type categories that specifically relate to a crime were generally cleared as the same call-type category (with the exception of “Assault”). Some of the overlap diminished, though, when accounting for crime report information. For example, we found reasonable overlap in classifications between the initial and cleared call-type categories for “Theft” and “Mischief.” However, once we included crime-type information obtained via crime report data in our assessments, these accuracy rates declined. The highest accuracy rates across all three classifications were observed for “Burglary” and “Robbery” and the lowest rate was observed for “Drugs.” This suggests that not all events that may appear as crime-related at their outset may actually regard crime and vice versa (e.g., there were also many crime reports for events that were not initially classified as crime-related call-types). It also suggests that not all events which are cleared as crime-related call-type categories result in crime reports or result in crime reports for different types of crime than initially classified. As alluded to above, this implies that miscommunication, misunderstanding, or even officer discretion could be inducing what appear to be misclassifications in the data, a point in which we interrogate in more detail below.

In sum, our results thus provide insight into the types of calls for service that police most frequently respond to as well as the relevance of call classifications for measurement error. For example, we find that most calls for service do not regard crime. Indeed, the most frequent call-type categories for this police agency were “Suspicious Circumstance,” “Alarm,” and “Disturbance.” When events do involve crime, classification accuracy can vary across crime-types. Our results also provide some indication for why measurement error may exist in these data and the role that different policing actors, including dispatchers and officers, play in producing such error at different parts of the policing process. Given the popularity of calls for service data in research (e.g., [39–44]), these findings exhibit several implications.

### Implications

Misclassified calls for service exhibit important implications for both operations and research. From an operational perspective, misclassified events can result in problematic decision-making by officers: they may prepare themselves for one type of event only to be presented with another, which could result in both under- and over-reactance, including in the use-of-force. Misclassifications could also delay the response of critical support resources, which in turn could pose risks to community members and officers alike. Call-types are a critical piece of information used by police as part of their deployment decisions, and so in order to effectively manage their operations, police must be able to rely on these classifications.

From a research perspective, misclassified events can also present many challenges. First, misclassified events can distort one’s understanding of the potential problems facing a community. For example, a cluster of misclassified events could lead researchers to believe that a problem may exist when one does not or that a different problem exists than the actual problem. This is particularly relevant when researchers use calls for service as a measure of crime, disorder, or other problematic behavior. In these cases, hot spots of alleged crime could actually reflect hot spots of misclassified events.

Second, misclassified events could impact one’s understanding of both the public’s demand on police as well as their willingness to cooperate with police, both of which are salient outcomes in criminological research. Given their flexibility, calls for service have become an important tool to assess how often and for what kinds of reasons the public request the police (e.g., for a discussion of how this applied to the COVID-19 pandemic in the USA, see [39]). If the public’s requests are not accurately captured via call classifications, the image of public demand produced via these data may distort reality and present challenges for inference. Moreover, changes in frequencies of calls for service are often used to assess the effects of social events (e.g., critical incidents, national sentiments, etc.) or changes in policing (e.g., new policies, organizational restructures, etc.) on the public’s willingness to cooperate with police. If events are misclassified such that they either over- or underrepresent categories of events, then the associated findings could mislead researchers. Indeed, non-crime-related requests, like the request for police to check the welfare of a person, are frequently salient in these research questions, and these requests are only captured via specific call-types in the CAD system. If these events are not accurately classified as such call-types, they are likely to be missed in the respective analyses.

#### Different types of data for different purposes

Our research also presents some broader implications for the use of different types of data in criminological research more generally. If the goal of the research is to assess the ways in which citizens self-define and interpret events that they report to the police, including what they believe are crimes, then it may be more appropriate to use calls for service data than crime report data (victimization data can also provide rich insight if analyzing events that are not reported to the police). However, as we have already now described, not all parts of calls for service data are created equally: initial call-types (which are “unverified” and generated by dispatchers) are much more flexible than cleared call-types (which are “verified” and generated by responding officers), despite both being included in the same calls for service data. And, both initial and cleared call-types are still more flexible than crime report data, which are more structured in nature and subject to the greatest formal filtering by police.

These strengths are not without weaknesses though. And, in light of the measurement error observed in calls for service data, the question becomes if these data should even be used in research. We suggest that they still can and should be used when the research question warrants them. Despite their limitations, calls for service data offer important insight into a plethora of policing-related topics. For example, they allow researchers to uniquely assess a much wider array of requests for police service than what crime reports allow, including non-crime-related and strictly service-oriented requests (which as we demonstrated comprise a substantial portion of police work). As described earlier, they also exhibit less formal institutional filtering, especially when examining the initial call-types of events. With each stage of the call handling process, there is more filtering of an event, and so by the time that it reaches the stage of a crime report (if it does), the event classification may be much more a reflection of the officer’s interpretation than the citizen’s interpretation.

With that in consideration, we recognize that if the goal of the research is to assess the prevalence of police-defined and reported crime, then it may be more appropriate to use crime report data. Nonetheless, crime report data may contain measurement error too. For example, there are political pressures and social factors which may incentivize the systematic misclassification of events in crime reports and/or the lack of crime reports all together (e.g., see [12, 31, 34]). As we highlighted above, officers may employ their discretion to not generate a crime report if they believe, for example, that it would not be in the public’s interest to pursue charges for the crime and/or if there is no means to investigate the crime. Crime reports also generate much administrative work for officers, including in the form of follow-ups, that some officers may wish not to pursue. In this way, crime report data may not reflect all crime events either, but rather the most operationally serious ones as defined by officers (i.e., the absence of a crime report does not always infer the absence of a crime). And this stacks on top of the related and well-documented challenge of assessing the “dark figure” of crime (e.g., see [45, 46]), which too complicates crime report data.

No source of data is perfect, and indeed, identifying the perfect source of data was not the intent of this research. Instead, we sought to explore dispatcher decisions to classify events as different call-types and the accuracy of such classifications as defined by responding officers. On this front, we propose several recommendations for future work involving calls for service data. First, we believe that it is important for researchers to carefully consider whether the initial or cleared call-type of an event most accurately reflects their intent of using calls for service data. As previously described, these classifications are different, sometimes in substance, but almost always in principle. Relatedly, we urge police agencies to include both the initial and cleared call-type information for all events in their open datasets to be used for research. This would allow for greater assessments of classifications moving forward. Finally, we encourage policymakers and practitioners alike to consider a possible tiered system for police call classification, which would permit dispatchers to indicate primary, secondary, and even tertiary call-types for each event. Doing so would allow for events to be better interpreted by other actors, which in turn could alleviate some of the challenges associated with event classifications.

### Limitations

The present research exhibits several limitations. First, and as noted in our methods, we acknowledge that our definition of accuracy may be contested. It is also possible that some scholars may argue that accuracy cannot or should not be measured among such a subjective process, which we too acknowledge, but nonetheless interrogate given the implications of using these “subjective” data. Consistent with this logic, we experienced much difficulty ourselves in grouping call-types into categories, and hence others may have categorized some call-types differently, which too could introduce error among the research process itself.

Second, we recognize that our analyses may imply some assumptions. For example, “misclassification” as a term may in principle refer to a problem, but in practice may not always infer a problem. An event could be correctly classified at the time it was generated with the information that was available at that time, but then re-classified to a different call-type at a later time after new information (which was not initially available) comes to the attention of police. In this case, the respective policing actor, most often the responding officer, can then make a more accurate classification in light of the newly obtained information. A classification can change as the information changes, suggesting that there could actually be *multiple* accurate classifications at *multiple* different points in time. From this perspective, it is reasonable to argue that misclassifications in these kinds of data may never truly be eradicated so long as there is room for subjective decision-making in the policing process. With that being said, we were unable to interrogate this possibility in much detail due to limited information provided by the current data.

Third, and relatedly, we used a linear approach as part of our analyses, such that we began with the initial call-type classification made by the dispatcher and then assessed accuracy moving forward. It would also be possible to use a reverse approach, where one could begin with the crime report classification and then explore classifications backwards. However, given the linear nature of police work, insofar that decisions are made based upon existing information available at the time of such decision, we believe that our approach was best suited for our particular research questions.

Fourth, we were unable to account for potential misclassifications made by dispatchers when they did not create a formal call for service in the CAD system. As argued by Lum et al. (2020), many calls to the police do not result in formal records, often because the call-taker is able to resolve the incident without the response of an officer. In these instances, it is possible that dispatchers may mistake a crime for no crime, and hence not create a formal record, but we have no means to assess the prevalence or nature of this type of error as part of the present research. Instead, we are only able to assess overlap in events that are actually documented via formal calls for service in the CAD system.

Fifth, we focused heavily on the role of dispatchers working in the capacity of call-takers as part of the present research. It is possible that other actors within the contact process, including dispatchers working in the capacity of radio-operators, may also impact classification accuracy (e.g., see [5]). Indeed, through their discretion, radio-operators may change call-types as they see fit in ways that could not be accounted for as part of the present research. There could thus be even more variation among the classification process than what we were able to assess, and such variation should form the subject of future research.

Sixth, and finally, we recognize that critical incidents, national sentiments, and seasonality (among others) could all affect call volume and reporting patterns at more aggregate levels (e.g., as discussed in the exchange between Desmond et al. [47, 48] and Zoorob [49]). Although the focus of the present research was on the classifications of formally documented calls for service from generation to closure, future examinations should also explore more social and seasonal trends in call patterns. It is possible that these more macro variables could systematically influence the substance and quality of calls for service data.

## Conclusion

As researchers, we understand the frustration that can be felt when attempting to use calls for service data as part of analyses, recognizing that the trajectory of a call from its outset to its closure is often unclear, providing little explanation to those who are examining the data without additional context or insight. As police dispatchers, however, we also possess firsthand knowledge about the rapid decision-making that takes place while generating calls for service, the often ambiguous and nebulous information provided by callers, and the piecing together of details that takes place while trying to accurately and reliably classify events. By combining our professional experience as police dispatchers with existing literature about police work and empirical analyses regarding call classifications, we contribute to this timely discussion about calls for service data and their implications for operations and research. Calls for service offer an important conduit by which officers and researchers can obtain insight into public requests for police service, however both officers and researchers must be mindful of their limitations when using them to inform their responses and analyses.
